# Supplementary material for: Efficacy of broflanilide (VECTRON T500), a new meta-diamide insecticide, for indoor residual spraying against pyrethroid-resistant malaria vectors
Source: Sci Rep. 2021 Apr 12;11:7976. doi: 10.1038/s41598-021-86935-3 (PMC8042056; doi:10.1038/s41598-021-86935-3)
Supplement: Supplementary file 1 — Supplementary Information. [file 41598_2021_86935_MOESM1_ESM.docx]

**Efficacy of broflanilide (VECTRON T500), a new meta-diamide insecticide, for indoor residual spraying against pyrethroid-resistant malaria vectors.**

**Authors:** Corine Ngufor^1, 2, 3*^ Renaud Govoetchan^1, 3^, Augustin Fongnikin^2, 3^, Estelle Vigninou^2^, Thomas Syme^1,3^, Martin Akogbeto^2,3^, Mark Rowland^1,3^

**Affiliations:**

^1^London School of Hygiene and Tropical Medicine (LSHTM), London, UK

^2^Centre de Recherche Entomologiques de Cotonou (CREC), Benin

^3^Panafrican Malaria Vector Research Consortium (PAMVERC), Benin

*corresponding author: Corine Ngufor

Email: corine.ngufor@lshtm.ac.uk

**Supplementary table information**

Table S1: Mortality (72hrs) of susceptible and pyrethroid resistant *An gambiae* strains exposed to broflanilide in CDC bottle bioassays

| Strain | Dose (µg/bottle) | 0 | 5 | 10 | 16.5 | 27.1 | 44.7 | 73.7 | 121.4 | 200 |
| --- | --- | --- | --- | --- | --- | --- | --- | --- | --- | --- |
| Susceptible *An gambiae* ss Kisumu | N | 170 | 92 | 163 | 198 | 168 | 150 | 144 | 143 | 144 |
|  | n dead (72h) | 12 | 10 | 158 | 112 | 108 | 138 | 144 | 143 | 144 |
|  | % Dead (72h) | 7 | 11 | 97 | 57 | 64 | 92 | 100 | 100 | 100 |
| Pyrethroid-resistant *An gambiae* sl Cove | N | 90 | 92 | 153 | 138 | 146 | 121 | 144 | 123 | 121 |
|  | n dead (72h) | 8 | 4 | 96 | 52 | 74 | 120 | 144 | 121 | 121 |
|  | % Dead (72h) | 9 | 4 | 63 | 38 | 51 | 99 | 100 | 98 | 100 |
